# Treatment injuries are rare in children's femoral fractures: Compensation claims submitted to the Patient Insurance Center in Finland

**DOI:** 10.3109/17453674.2010.533927

**Published:** 2010-11-26

**Authors:** Sauli Palmu, Reijo Paukku, Jari Peltonen, Yrjänä Nietosvaara

**Affiliations:** ^1^Children's Hospital, Helsinki University Central Hospital, Helsinki; ^2^Orton Orthopaedic Hospital, Orton Foundation, Helsinki; ^3^Orto-Lääkärit, Medical Center, Helsinki; ^4^the Patient Insurance Center, Helsinki, Finland

## Abstract

**Background and purpose:**

The current treatment for femoral fractures in children is mostly operative, which contrasts with treatment of other long bone fractures in children. We analyzed treatment injuries in such patients in Finland in order to identify avoidable injuries. Our other aims were to calculate the incidence of these fractures and to describe the treatment method used.

**Methods:**

The Patient Insurance Centre (PIC) provides financial compensation of patients who have sustained an injury in connection with medical care. We retrospectively analyzed incidence, treatment methods, and all compensation claims concerning treatment of femoral fractures in children who were 0–16 years of age during the 8-year period 1997–2004.

**Results:**

The incidence of childhood femoral fractures in Finland was 0.27 per 1,000 children aged < 17 years, and two-thirds of the patients were treated operatively during the study period. 30 compensation claims were submitted to PIC during the 8-year study period. The compensation claims mainly concerned pain, insufficient diagnosis or treatment, extra expenses, permanent disability, or inappropriate behavior of medical personnel. Of the claims, 16 of 30 were granted compensation. Compensation was granted for delay in treatment, unnecessary surgery, and for inappropriate surgical technique. The mean amount of compensation was 2,300 euros. Of the injuries that led to compensation, 11 of 16 were regarded as being avoidable in retrospect.

**Interpretation:**

The calculated risk of a treatment injury in childhood femoral fracture treatment in Finland is approximately 2%, and most of these injuries can be avoided with proper treatment.

The reported incidence of femoral fractures in childhood varies between 0.22 and 0.33 per 10^3^ children (Lyons et al. 1999, Bridgman and Wilson 2004). Femoral fractures represent 1–2% of all fractures in chidren and adolescents (Landin 1997, Lyons et al. 1999, Hedin 2004). Together with forearm and tibial fractures, they are the most common childhood long-bone injuries (Salem et al. 2006).

For a long time, femoral fractures have been treated by traction and/or casting. More recently, surgery has gained popularity (Yandow et al. 1999, Hedin 2004, Bopst et al. 2007). Hedin (2004) has proposed a treatment protocol for femoral fractures in children whereby operation is the preferred option in children over 3 years of age.

With nonoperative treatment, complications include malunion, nonunion, and skin lesions. In addition to these, operative treatment can lead to nerve injuries, infections, or pain and irritation at the site of incision (Narayanan et al. 2004, Wall et al. 2008).

We have reported treatment injuries in children's lower leg fractures in an earlier study (Palmu et al. 2009). There have not, however, been any studies on treatment injuries of femoral fractures in children. In this study we explored what kind of treatment injuries occur and we identified avoidable injuries. We also calculated the incidence. In this paper we also describe the method of treatment of femoral fractures in children in Finland.

## Patients and methods

The Patient Insurance Center (PIC) in Finland grants compensation to patients who have sustained injuries associated with medical care without having to prove any treatment to be faulty. According to the Finnish Patient Injuries Act, a compensatable treatment injury has occurred if an experienced medical professional would have proceeded in a different manner and thus avoided the injury. The patient information and data concerning compensation claims for femoral fractures in children during the study period came from the registers of the PIC. In their claims for compensation, parents provided demographic data and a description of the injury. The PIC medical adviser evaluated whether a compensatable treatment injury had occurred, based on the medical records. The PIC made the final decision regarding compensation.

An independent observer (a consultant pediatric orthopedic surgeon (RP) who was not involved in patient treatment or in handling of claims) retrospectively analyzed all patient claims (n = 30) and decisions concerning treatment during the study period (1997–2004), with re-evaluation of patient treatment files, statements of PIC experts, and decisions about compensation. Age, sex, and type and location of the fracture were recorded. Trauma energy was graded as high (traffic accident, fall from a height > 6 m), moderate (sporting injuries), or low (falling on level ground), and mode of treatment, complications, and permanent sequelae were assessed along with reasons for the claim and for the compensation. Information concerning the amount of compensation for these patients was provided by PIC, and the number of avoidable treatment injuries was estimated.

The annual incidence of femoral fractures in children was calculated using registry data and the method of treatment was analyzed in retrospect using the registers of the National Institute of Health and Welfare.

The statistical analysis was performed using SPSS software version 16.0. 95% confidence interval (CI) for incidence was calculated using the Poisson distribution.

## Results

During the 8-year study period (1997–2004), the PIC received 30 compensation claims concerning femoral fracture treatment in children. The mean age of these patients treated in healthcare centers (n = 6) was 3 (0–11) years, and it was 11 (0–16) years in hospitals of different kinds (n = 24). There were no open fractures, but there were 3 pathological fractures: 2 children had simple bone cysts and 1 had osteogenesis imperfecta. 1 child suffered multiple injuries after a fall from the sixth floor (Table 1).

**Table 1.** Characteristics of the 30 claims for compensation submitted to the PIC

Age	Injury	Frac-ture ^a^	Treatment institution ^b^	Primary treatment ^c^	Complication ^d^	Compensation claim	Compensation, reason	Avoidable
0.1	Fall < 1 m	S	HC	T		Delay in diagnosis	No, no effect on outcome	Yes
0.5	Fall < 1 m	M	HC	None		Delay in diagnosis	No, no effect on outcome	No
0.6	Fall on level	S	CH	None		Delay in diagnosis	Yes, inadequate clinical examination	Yes
1,2	Fall on level	S	CH	T + C		Delay in diagnosis	Yes, fracture dislocated due to delay	Yes
1.6	Fall on level	S	HC	C		Delay in diagnosis	No, no effect on outcome	Yes
1.8	Fall on level	S	HC	T	SU	Inadequate casting	Yes, inadequate cast padding	Yes
2.0	Child abuse?	S	CH	T		Pain and suffering	No, satisfactory treatment	No
2.1	Child abuse?	S	CH	T	SU	Inadequate treatment	No, ulcer not caused by treatment	No
3.2	Fall > 15 m	M	UH	T	SU	Inadequate treatment	Yes, wrong treatment method	Yes
4.3	Ice hockey	S	UH	ST		Delay in treatment	No, fracture not visible in radiographs	No
4.5	Playground	S	CH	T + C		Angular deformity	Yes, angular deformity	No
4.7	Sledding	M	HC	None		Missed diagnosis	Yes, fracture missed on radiographs	Yes
5.6	Fall on level	S	UH	FIN		Pain and irritation	No, satisfactory treatment	Yes
5.7	Child abuse	S	CH	SF	Inf	Infection	Yes, unreasonable infection	No
6.9	Sledding	S	HC	None		Delay in diagnosis	Yes, no primary radiographs	Yes
7.6	Downhill skiing	S	CH	FIN		Growth plate injury	No, caused by primary injury	No
8.2	Fall 4 m	S	CH	ST	Inf	Infection	Yes, unreasonable infection	No
8.2	Sledding	S	CH	T + FIN	SU	Inadequate treatment	No, satisfactory treatment	No
8.8	Bicycle	S	UH	FIN	BI	Burn injury	Yes, inadequate use of diathermia	Yes
9.2	Downhill skiing	P	CH	C		Growth plate injury	No, caused by primary injury	No
10.2	Ice hockey	S	CH	C		Inadequate treatment	No, satisfactory treatment	No
10.2	Soccer	M	UH	SF		Pain and irritation	No, satisfactory treatment	No
11.9	Fall on level	M	CH	SF		Inadequate treatment	Yes, inproper positioning of screws	Yes
14.0	Soccer	P	CH	None		Inadequate treatment	No, satisfactory treatment	Yes
14.4	Fall on level	M	UH	SF	Inf	Infection	Yes, unreasonable infection	No
15.4	Riding	S	UH	RIN	JSD	Pain and irritation	Yes, damaging joint surface by RIN	Yes
15.4	Bicycle	S	CH	RIN		Growth disturbance	Yes, wrong treatment method	Yes
15.4	Motorcycle	S	UH	FIN		Pain and irritation	No, normal treatment	No
15.6	Motorcycle	S	CH	FIN	PA	Inadequate treatment	Yes, nail removal too early	Yes
16.8	Motorcycle	S	DH	RIN	PA	Inadequate treatment	Yes, diagnosis of PA was delayed	No

^a^ M: metaphysis, P: physis, S: shaft,

^b^ CH: central hospital, DH: district hospital, HC: healthcare center, UH: university hospital

^c^ C: cast, FIN: flexible intramedullary nailing, RIN: rigid intramedullary nailing, SF: screw fixation, ST: skeletal traction, T: traction

^d^ BI: burn injury (caused by inadequate use of diathermia), Inf: infection, JSD: joint surface damage (caused by intramedullary nailing), PA: pseudo-arthrosis, SU: skin ulcer

Primary treatment (16 operative, 6 skin traction, 3 casting, and 5 with no initial treatment) was given in healthcare centers (6 patients), a district hospital (1 patient), central hospitals (15 patients), and university hospitals (8 patients). The operative treatment of 16 of the 30 patients consisted of intramedullary nailing in 8 children (4 elastic and 4 rigid nails), skeletal traction in 4, screw fixation in 3, and plate osteosynthesis in 1 child. The nonoperative treatment consisted of skin traction continued by circular casting in 6 children, hip-spica casting in 2, and circular casing only in 1 child. Of the 5 children with no initial treatment, 4 were later treated by casting. The mean age of children treated nonoperatively was 4 years and it was 11 years in children treated operatively. Complications occurred in 11 of the 30 children (Table 1).

The claims for compensation were based on pain (n = 20), insufficient diagnosis or treatment (n = 17), extra treatment expenses (n = 9), permanent disability (n = 7), and inappropriate behavior of medical personnel (n = 2). In 17 cases, there were claims for more than 1 issue. Of the 30 claims for compensation, 16 were granted. Compensation was granted for 13 treatment injuries and 3 infections. The treatment injuries involved delay in treatment of 3 children, unnecessary operation in 2, inappropriate surgical technique in 2, and other reasons in 5 children. The delay occurred in diagnosis in 2 children and in detecting nonunion in 1 child. The surgical techniques considered to be inappropriate in retrospect were plate fixation of a subtrochanteric fracture and inadequate intramedullary nailing leading to joint surface damage. All 3 infection injuries were related to operative treatment and they were considered to be unreasonably serious.

The PIC granted an overall sum of approximately 42,000 euros as compensation to the patients. The average compensation granted was 2,300 euros. Compensation was granted for permanent sequelae (14,200 euros), for pain (13,700 euros), for cosmetic reasons (9,600 euros), and for other reasons (4,200 euros). The PIC estimated that approximately 32,000 euros would still be paid to the patients.

Of the 16 injuries that were given compensation, in retrospect we regarded 11 of them as being avoidable. The injuries we regarded as being unavoidable were a nonunion in a child with a broken intramedullary nail, 3 postoperative infections, and a malunion after casting. In the latter child, we did not agree with the PIC compensation for an angular deformity in a 4-year-old child which would most likely have remodeled.

During the study period, the mean total population of Finland was 5.2 × 10^6^ inhabitants; of these, 1.1 × 10^6^ were children. The calculated annual incidence of childhood femoral fractures was 0.27 per 10^3^ (CI: 0.10–0.29). The treatment method was operative in two-thirds of cases during the study period (Table 2). The calculated risk of sustaining a patient injury in treatment of childhood femoral fractures in Finland during this period was 2.2%.

**Table 2.** The method of treatment of 1,389 childhood femoral fractures in Finland during 1997–2004 according to national registry data

Method	n
Cast immobilization in situ	142
Manipulation + cast immobilization	229
Skin traction	29
Internal fixation	762
intramedullary nail	616
screw fixation	87
plate osteosynthesis	59
Skeletal traction	143
External fixation	50
Unspecified operative treatment	0
Reoperation	14

## Discussion

Femoral fractures constitute 2% of all fractures in children. According to Lyons et al. (1999), the incidence is 0.32 per 1,000 children. Despite its rarity, femoral fracture is the commonest children's trauma to end up with hospitalization (Loder et al. 2006). The national incidence in Finland (0.27 per 1,000) based on register data is in accordance with earlier reports. We believe that this figure is reliable since, with few exceptions, these children are hospitalized.

The number of complications in all children's femoral fractures treated in Finland that are reported here is most likely an underestimate, since we only evaluated the ones that led to filing of a compensation claim. Most of the treatment injuries were regarded in retrospect to be avoidable with more careful clinical practice: careful clinical examination and follow-up including skin examination and radiography. The reasons for unavoidable injuries were mostly infection-related. In previous studies operative treatment has led to minor complications such as pain or superficial infections. More severe complications include deep infection, malunion, and neurological deficits (Narayanan et al. 2004, Wall et al. 2008). Narayanan et al. (2004) also suggest that the complications are potentially avoidable. In our series, one-third of the patients suffered from complications. These were similar to those reported earlier.

The average amount of compensation was 2,300 euros. The most common reasons for compensation claims were excessive pain and/or insufficient diagnosis or treatment. These are matters that could be avoided with normal clinical practice. Although the amount of compensation was generally low, this extra cost and unnecessary suffering of the children could be avoided.

The treatment method for femoral fractures in children varies. According to the recommendation of Buckley (1997), children under the age of 6 should be treated with spica casting, those from the age of 10 like adult patients, and children from 6 to 10 with either casting or by operative means. Hedin (2004) on the other hand, recommended traction and spica casting only for children under the age of 3, with others being treated operatively. According to Finnish national register data, two-thirds of children with femoral fractures were treated operatively. The primary treatment method in the children described here was nonoperative in 17 patients and operative in 13 patients. There was a difference in the mean ages of these patient groups: 4 and 11 years, respectively, which is in line with recommendations. The parents of the patients who were treated operatively filed less claims for compensation than those treated nonoperatively. This may mean that there was more satisfaction with treatment.

In conclusion, most femoral fractures in children are treated operatively in Finland. Most of the treatment injuries can be avoided.
